# Botany in Its Relations with Medicine, No. 1

**Published:** 1877-10

**Authors:** W. T. Grant

**Affiliations:** Atlanta, Ga.


### BOTANY—IN ITS RELATIONS WITH MEDICINE—No. 1.

BY W. T. GRANT, M.D., ATLANTA, GA.

Some years since, I published several articles on the subject of botany in connection with medicine, but left it incomplete. I now resume my pen, although I cannot be sure that I can yet say all that I should like in advocacy of the more general study of the science of botany by the profession.

It is told that the Pope of Rome at one time said that he was determined that the devil should not have all the good music, and he at once adopted a number of secular airs, and thereby greatly improved the church music. In a similar spirit we should say that the botanics and eclectics, and other wandering sheep of the medical fold, shall not appropriate to themselves the entire field of botany.

By-the-by, this illustration is not exactly appropriate, as when the Pope adopted secular music, he was, in fact, appropriating to his use something which did not belong to him; whereas, in our case, we are but claiming back our own property, which these naughty brethren have stolen and are trying to make a claim to. The fields and forests were originally, and in the early times, the only source from which the profession of medicine drew its material supplies with which to combat disease. And it by no means follows that, because chemistry offered us a large additional supply of medicines which were accepted, that we thereby gave up our claim upon the fields and forests. By no means. Yet it would seem that some think so, as these erring brethren, the botanics and eclectics, have taken possession of this source, and while attempting to set up house for themselves, seem disposed to warn us off *their* provinces, and call us "mineral doctors," etc., etc.

The entire kingdoms of nature belong to us of right—the animal, the vegetable and the mineral; and we reject nothing whatever that will aid in conquering disease. Hence, I am disposed to denominate ourselves as *all*-opathics, instead of alopathics.

In the articles above mentioned, I took the position that *every plant had some one character, or combination of characters, which would indicate the fact that it possessed medicinal virtues,* and that by means of the same or other similar characters, it would be possible to tell at sight what virtue it possessed—whether emetics, cathartics, or any other. After elucidating this proposition, I then insisted that there were not careful observers enough in the fields taking notes. These special characters or indices by which we were to determine the medicinal value of plants, are not now known, but will have yet to be sought for and determined. And while we have excellent and superior advantages, as well as fine botanists, we can never attain this desirable end until the two are combined in one. If every physician was also a practical botanist, no one could predict the immense advance which in a few years would be made. I therefore urge that every medical college should have a chair of botany, for training the students in this branch. I do not mean that this should be a mere appendage to the chair of materia medica, but a separate and distinct professorship. It would not occupy any more time, and very soon after the course begun, I venture the assertion that the interest which the study would develop, would far more than compensate the students for the additional application needed. I am quite sure that the exceptions would be quite rare among students for them to think it any added labor. After they have mastered the elements (which, with a competent teacher,) would be a small matter, they would pursue the study with increasing pleasure, and the result would be that every physician who left your schools would be but another observer in the field, ready and anxious to correct old errors, ascertain new facts, and forward the interests of his profession.

How many physicians are there now in Georgia who can take any of the medical journals of the day and tell whether the plants therein recommended grow in his vicinity? The botanical names are a "sealed book" to nine-tenths of them, and if they should want to use any plant thus recommended, they would probably send a distance for it, although it might be abundant at their doors.

The science of chemistry has been assiduously cultivated by competent men for the past quarter of a century, and as a result, we have a large number of new compounds and preparations introduced to our notice as valuable remedies. If the same attention was given to botany, a like consequence would result. The resources of our forests are unknown, and are almost illimitable. Even with the limited knowledge which I possess of the subject, I believe that I can go into the woods of Georgia and get plants with which I can produce almost any given or desirable effect. The true sequence of matters should be, botany to point out the plant, and then for chemistry to determine its active principles. Botany should lead, but there are no botanists.

The maruta cotula is reputed to possess superior virtues in vesical complaints; but it is not used, because very few know that the weed, which grows by millions over the land, in every nook and corner, and commonly called mayweed, is the maruta cotula. The same may be said of the daucus pucillus; also of salvia lyrate, and a host of other plants which grow all over the country. I believe that I could cover two pages of this journal with simply the *names* of valuable medicinal plants, which are to be found within the limits of our State. And even of the older and better known plants, how many physicians would be able to recognize them? We have quite a number among us. Podophyllum, peltatum, datura stramonium, chimaphila, maculata, the gentians, the lobelias and many others, are our near neighbors, and we have scarcely to do more than reach forth our hand to get them fresh and pure.

I had a thought, some time since, of making a collection of our medical plants, and then prepare an essay, giving briefly their various virtues. I then expected to read the essay before some medical society, and exhibit each plant as an account was given of it, but circumstances interrupted the execution of my plan.

I think that there is a rich vein of wealth that will yield good returns, in what is called domestic practice, in which nothing but plants is ever used by the good old matrons of the land. But none need delve here except the medical botanist. Many valuable remedies are now buried under their common or domestic names, which, if examined by the botanist, could take their true and proper place. By ascertaining its proper name, its various relations are at once seen, and a reason can be easily found why it should be valuable.

I am not, in any sense, a botanist, and I only insist that botany should be taught in our schools, because I know so well what great benefit will result. It will then put this branch of our materia medica on a par with chemistry.

I may be allowed to say that the botanic school have done but little to advance our knowledge of the vegetable kingdom, but the Eclectics are making good progress. Of course it will require time to sift out the really valuable remedies from their legion of panaceas, but that will not detract from the merit of any really valuable discoveries they may make.

